# The Treatment of Lachrymal Obstruction
1A Paper read at a meeting of the South-Western Ophthalmological Society, November 24th, 1925.


**Published:** 1926

**Authors:** A. E. Iles

**Affiliations:** Surgeon, Bristol Eye Hospital; Ophthalmic Surgeon, Bristol General Hospital


### THE TREATMENT OF LACHRYMAL OBSTRUCTION.[1]

BY

A. E. Iles, O.B.E., M.B., F.R.C.S.Eng.,
*Surgeon, Bristol Eye Hospital;*
*Ophthalmic Surgeon, Bristol General Hospital.*

In bringing forward this subject for discussion I have been guided by the fact that many ways of treatment have been devised for its relief, upon which widely divergent views are held.

In general, surgical principles aim at restoration of the lumen to a blocked canal (if the canal be an essential one) or formation of an alternative route. It is with this premise that I set out the remarks in this paper.

In order to treat cases of lachrymal obstruction efficiently we must determine accurately the cause of its occurrence. Glancing first of all at the anatomical relations and structure of the canal, we find it lying first in soft tissues and then later surrounded by an unyielding bony canal. Physiologically the canal can be divided into two parts by means of its epithelium. In the first part, as far as the sac, it is a stratified pavement epithelium, and in the latter part it consists of a double layer of cylindrical epithelium. In this latter part of its course the wall of the canal is very freely supplied with vascular tissue, especially veins. There is also a considerable amount of adenoid tissue present. The presence of the adenoid tissue and the

[1] A Paper read at a meeting of the South-Western Ophthalmological Society, November 24th, 1925.

veins implies that obstruction may be readily caused by engorgement of the veins from nasal mischief, or by increase in the adenoidal element present.

When we look upon the number of cases of acute conjunctivitis in our out-patient and private practice, and recollect that a good deal of the secretion finds its way into the tear passages, we ask ourselves whether we ought to be more thorough in the treatment of the inflamed conjunctiva. I myself have frequently failed to get, by cultural methods, the organism causing the trouble. The researches of Lindner, and the recent confirmation of his work by Howard,[1] on the behaviour of pathogenic organisms in relation to the conjunctival epithelium, explain this difficulty. They point out that gonococci, pneumococci, the Koch-Weeks and influenza bacilli are only present in the discharge for a short while, then they are to be found in turf-like processes on the epithelium, especially at the cell borders (first on the bulbar and then on the palpebral conjunctiva). Later they penetrate the cell envelope, the gonococcus to the deepest extent, and by their ectotoxins stimulate the subjacent layers of epithelium, so that marked proliferation takes place. This process is often carried to excess, so that the epithelium is thicker than before. Phagocytosis by the epithelial cells takes place (leucocytosis in these inflammations is very limited). Finally, the surface epithelium comes off in patches. In this way organisms may find their way into the lachrymal canal, attach themselves to the epithelium, and stimulate the deeper layers of epithelium so that the mucous membrane becomes thicker and the lumen of the canal narrower than normal. This applies chiefly to the upper part of the canal. In order to attack the organisms once they have invaded the cells themselves it is necessary to use lavage containing a body which has the property of attacking the invaders in the cells. With this object in view I have been using mercurochrome, 1 per cent, and have found it efficacious in clearing up pus rapidly, without doing harm to the tissues. Substances which cause necrosis, such as nitrate of silver, should not be used.

### LACHRYMAL OBSTRUCTION IN INFANTS.

In these a mucocele may be present or there may be just continual watering of the affected eye. This depends on the fact that in very many instances the lachrymal canal does not become patent until shortly after birth, the obstruction being due either to a very thin membrane or to a plug of epithelium at the nasal orifice. Occasionally there is a spontaneous cure. In others I have effected a cure by means of a probe passed under an anæsthetic. Crigler[2], however, points out that since these mucoceles are not inflammations of the sac but rather infections of the retained products, a cure can be usually effected in the following manner. The essentials are that (*a*) the mucocele be as full as possible, (*b*) the patient be held as for examination of the eyes, *i.e.* between the knees, (*c*) the thumb is placed so that regurgitation from the sac to the conjunctiva through the punctum be prevented then rotated so that it is over the distended sac. Firm, abrupt pressure is then applied to the sac ; this forces the fluid from the sac down the canal, thus carrying away the obstruction.

### LACHRYMAL OBSTRUCTION IN ADULTS.

### (*a*) Obstruction without a Mucocele.

(i.) *At the punctum.* Dilatation with a dilator is the best treatment for these cases, as its use does not alter the capillary action by means of which the tears are conveyed to the lachrymal canal. If an incision has to be made, it is important that the gutter lie on the palpebral side, so that the furrow is in apposition to the globe and not on the lid margin.

(ii.) *Obstruction at the entry to the sac.* This may be brought about by a plug of mucus, actual engorgement of the mucous membrane thus giving rise to a narrowing of the lumen ; also by a stricture at the point where the canaliculus enters the sac.

In these cases it is my practice first of all to dilate the punctum, then to syringe in order to ascertain whether fluid will pass or no ; then I gently pass a large bulbous-ended probe to find out where the stricture is situated. It is of the greatest advantage to pass a little adrenalin into the canal to the site of obstruction ; this renders the parts less congested and instrumentation is made more easily. The advantage of a large probe is that it fully dilates the passages and therefore lessens the danger of a fold of mucous membrane being pushed in front of it. Access to the sac being gained, the probe is left in situ for ten minutes, and in the majority of cases the probe, which was tightly gripped at first, will be found to be much freer, and a larger one can then be passed. As a rule I leave a little mercurochrome in the sac.

Styles were formerly employed for this class of case, but owing to their use in unsuitable cases the practice has been discontinued.

In these cases, also, where there was a stricture in the neighbourhood of the entrance into the sac, slitting of the canaliculus has been practised, combining with the use of Weber's knife to divide the stricture. This treatment, with the use of probes, is impracticable if the patient is unable to come regularly at gradually lengthening intervals for nine months to a year. A great deal of the non-success which attended this mode of treatment was due to the fact that the patients were not made fully aware of the time required at the outset, and, in consequence, directly the condition was relieved to a great extent, considered themselves cured, whereas, as in stricture of the urethra relieved by sounds, regular attendance is required for a considerable time.

(iii.) *Obstruction below the sac.* In this type of case the services of the rhinologist should be sought, as it is of no avail to treat obstruction from the eye when the cause of the trouble is in the nose. My colleague, Mr. Wright, tells me that in 100 consecutive cases of lachrymal obstruction he found nasal trouble in 20 per cent.

In these cases, the sac should first be syringed out ; then a probe should be used in the manner described above. Very frequently one can feel the tip of the probe engage in a small depression, and if gently held there for a little while, in many cases it will be found to slip past the obstruction. Great gentleness and good anæsthesia are required in these cases. The patient then does not object, and there is also less danger of any untoward accident occurring. If this is done and the probing fails to be successful no harm ensues, while on a subsequent occasion a probe will often pass.

In one case of mine, a patient with healed lupus of the nose when I first saw her had complete obstruction one side and a very small passage on the other. At the first attempt I was only able to pass a probe on the one side, but at the second visit I was able to pass one on each side. Now the passages will take the largest probe quite easily. I have not had the necessity to pass a probe on the one side for more than eight months ; the tighter side is responding to treatment, and she is coming to me at gradually lengthening intervals, at present every four months.

(*b*) **Obstruction with a mucocele.**

The infantile cases have been described above.

In adults, owing to obstruction to the passage from without, or within, the sac becomes dilated. Now when this occurs and has been present for some time, the walls of the sac become thickened and the sac becomes a definite cavity : treatment of the obstruction is made more difficult owing to the fact that the presence of the enlarged sac has probably altered the relations of the upper and lower orifices, and inflammation is readily kept up by the stagnation of inflammatory products therein. In this type of case it is essential to keep the sac as free from micro-organsisms as possible.

As a rule operative interference becomes necessary in these cases and the methods that are in vogue are—

(1) Intranasal dacryocystotomy. (West.)
(2) Extranasal dacryocystotomy. (Toti.)
(3) Extirpation of the sac.

To my mind the first two operations carry out surgical principles more thoroughly than the third, in that they both endeavour to provide for the tears an alternative route. Most of these cases of lachrymal obstruction occur in women. One patient of mine when she was very near full time, came to me with a large mucocele. She was anxious not to have anything radical done until after the confinement. I saw her again a week later when she complained of discomfort in the swelling which was distinctly larger. To tide her over I decided to aspirate the sac with a hypodermic needle ; drawing forth distinctly turbid fluid, I left behind in the sac a little mercurochrome. I saw her again about one month later ; the sac had greatly diminished in size, the contents being now practically clear, and they could be expressed easily into the conjunctival sac.

Which operation is the best for each type of case ? Those cases in which the sac is shut off both above and below and there is no excess of epiphora are usually suitable for excision of the sac, which is also indicated in those cases where there is an ocular complication such as a corneal ulcer, and before operation for cataract where the sac is definitely infected.

When the sac is obstructed below the formation of a fresh opening into the nose is of great value, especially in those patients who have definite epiphora. Fraser, of Edinburgh, has recently published a further 42 cases in which he has performed this operation ; he reports 66 per cent. cures, and 25 per cent. recurrences, some failures. I myself favour the intranasal as against Toti's operation for the reason that if abnormalities are met with in the outer wall of the nose they can be dealt with more easily.

This is well illustrated in a case of mine ; a patient who had been treated with syringing and occasional probing for some time with success developed later on a complete block. I suggested that she should see a rhinologist, and Mr. Wright and I decided to perform West's operation. At operation there was found an enlarged air-cell in the region of the nasal incision ; this had to be opened out before the outer wall of the nose was exposed. When this was opened and a large piece of sac removed good drainage was secured. The obstruction in this case was due to the growth of the air-cell causing distortion of the tear passages. In another case in which West's operation had been performed the new opening closed up after a short while, but later the fluid when syringed through passed through the normal opening in to the inferior fossa of the nose, the new opening giving rest to the inflamed passages.

There are cases in which the obstruction is caused by removal of the sac. These cases are very unfortunate both for the surgeon and the patient. To relieve the condition excision of the lachrymal gland has been advised and performed ; this, to my mind, is unsound, for the conjunctival sac is deprived of its tears to wipe away undesirable objects and the eye becomes dry, a very unfortunate thing if the patient has to go to the tropics.

Morax[3] has recently described an operation devised to reform the tear passages ; a graft is made from the inner side of the arm and wrapped round a paraffin cylinder ; the middle meatus of the nose is opened from outside, the mucous membrane incised and sutured forwards, the graft being placed from the commissure to the nose.

### Acute Dacryocystitis.

If the patient is first seen when the abscess has already formed the best way is to incise the swelling, clean out the pus and pack with mercurochrome. In this way the inflammation rapidly subsides. In cases where the sac is only tender and there is no way either into the conjunctiva or nose, aspiration of the sac by means of a hypodermic needle and insertion of mercurochrome, 1 per cent., should assist in resolution of the inflammation. I have treated eleven cases in this way ; in eight the condition cleared up satisfactorily, while in the other three resolution was hindered by the skin being involved in the inflammatory process.

### Summary.

1. In early cases treatment should aim at prevention of the formation of a mucocele.

2. Energetic treatment of acute conjunctivitis is advisable.

3. Intranasal causes must be treated where they exist.

4. In infantile cases Crigler's method is advisable ; if that fails, an anæsthetic and a probe are needed.

5. In obstruction without enlargement of the sac, careful syringing, dilating and probing should be used. In these cases the intranasal operation has not been found to be a success. If probing fails then resort can be had to other means, one of which Morax has suggested.

6. Where a large sac exists with upper and lower openings obstructed and no epiphora, excision of the sac is indicated. This also should be undertaken when there are complications such as a corneal ulcer, and before the extraction of a cataract.

7. When the lower opening is blocked intranasal dacryocystotomy will in many instances afford relief. Those cases in which the upper opening is obstructed are not, in my opinion, favourable cases.

8. Restoration of the tear passages in the manner described by Morax appears to be a valuable proceeding, inasmuch as it aims at restoration of an impervious channel and lessens lachrymation.

9. Examination of the nose will frequently give valuable information, and will help in those cases where syphilis is a factor.

In conclusion, it appears that no one line of treatment nor any particular operation is suitable in every case. Owing to the very many differing conditions which may present themselves on examination, also to the differing causes giving rise to the obstruction, each case should be judged on its merits and treated accordingly.

The temperament and social condition of the patient have also to be carefully studied, also whether the patient will be able to attend regularly or no.

REFERENCES.

[1] Howard, *Amer. Jour. of Ophthalmology,* Dec., 1924, p. 208.

[2] Crigler, *Jour. Amer. Med Assoc.,* 1923, p. 231.

[3] Morax, *Annales d'Oculiste,* March, 1925.

